# Supplementary material for: Spatial disparities in the mortality burden of the covid-19 pandemic across 569 European regions (2020-2021)
Source: Nat Commun. 2024 May 18;15:4246. doi: 10.1038/s41467-024-48689-0 (PMC11102496; doi:10.1038/s41467-024-48689-0)
Supplement: Supplementary file 1 — Supplementary Information [file 41467_2024_48689_MOESM1_ESM.pdf]

Spatial Disparities in the Mortality Burden of the Covid-19 pandemic across 569 European Regions (2020-2021)

Online supplementary appendix A

Supplementary Table 1: Regional division, sources, data information and adjustments by country

Country	Spatial units	Source	Period	Upper age limit	Adjustments/comments
Austria	9 NUTS 2 units (‘Länder’)	Statistics Austria, Eurostat	1990–2021	Deaths: 115<br>Pop.: 95 (after 2001:100)	–
Belgium	11 NUTS 2 units (‘provincies’)	Belgian Statistical Office	1993–2021	Deaths: 100<br>Pop.: 100	–
Czechia	14 NUTS 3 units (‘kraje’)	Czech Statistical Office	1996–2021	Deaths: 95<br>Pop.: 95	–
Denmark	11 NUTS 3 units (‘landsdele’)	Statistics Denmark	2008–2020	Deaths: 90<br>Pop.: 90	–
Estonia	1 NUTS 2 unit	Statistics Estonia	1989–2021	Deaths:100<br>Pop.: 85	–
Finland	4 NUTS 2 units (‘Storområden’)	Statistics Finland	1972–2021	Deaths: 95<br>Pop.: 95	We merged Åland Islands to South Finland
France	95 NUTS 3 units (‘départements’)	INSEE	1970–2021	Deaths: 105<br>Pop.: 105 (after 2016: 95)	Non-European areas excluded; north and south of Corse merged to maintain consistent time series
Germany	96 ROR (‘Raumordnungs-regionen’)	Statistical Offices of the German Länder	1992–2021	Deaths: 90<br>Pop.: 90	Harmonized to apply current territorial administrative division (as of Dec 2022) to the whole study period and to eliminate the Census 2011 break; 400 NUTS 3 units (‘Kreise’) aggregated to 96 ROR units according to classification of BBSR (2017) [36]
Hungary	8 NUTS 2 units (‘tervezési-statisztikai régiók’)	Hungarian Central Statistical Office, Eurostat	2001–2021	Deaths: 90<br>Pop.: 90	–

Iceland	1 NUTS 2 unit	Human Mortality Database, Eurostat	1970–2021	Deaths: 110 (2021:100)<br>Pop.: 110 (2021:100)	–
Ireland	1 NUTS 1 unit	Human Mortality Database, Eurostat	1990–2021	Deaths: 110 (2021:100)<br>Pop.: 110 (2021:100)	No subnational division due to data availability issues
Italy	92 NUTS 3 units (‘province’)	ISTAT	1995–2021	Deaths: 100<br>Pop.: 100	We merged the following regions to maintain a consistent time series: 1) Biella + Vercelli, 2) Novara + Verbano, 3) Como + Lecco, 4) Milano + Lodi + Monza + Brianza, 5) Rimini + Forli-Cesena, 5) Firenze + Prato, 6) Cagliari + Medio Campidano + Carbonia-Iglesias + Ogliastra + Oristano + Nuoro, 7) Sassari + Olbia-Tempio, 8) Foggia + Bari + Barletta, 9) Fermo + Ascoli-Piceno, 10) Crotone + Vibo Valentia + Cantanzaro.
Latvia	1 NUTS 2 unit	Official Statistics Portal Latvia	1990–2021	Deaths: 100<br>Pop.: 100	–
Lithuania	2 NUTS 2 units	Eurostat	2001–2021	Deaths: 85<br>Pop.: 100	–
Luxembourg	1 NUTS 3 unit	Human Mortality Database	1996–2021	Deaths: 110<br>Pop.: 110	–
Netherlands	12 NUTS 2 units (‘provincies’)	Statistics Netherlands	1990–2021	Deaths: 100<br>Pop.: 90 (after 2001: 100)	–
Norway	7 NUTS 2 units (‘landsdeler’)	Statistics Norway	2000–2021	Deaths: 100<br>Pop.: 105	Harmonized to apply current territorial administrative divisions; we excluded remote islands Svalbard and Jan Mayen
Poland	73 NUTS 3 units (‘podregiony’)	Statistics Poland	2006–2021	Deaths: 90<br>Pop.: 100	–
Portugal	5 NUTS 2 units (‘regiões’)	National Institute of Statistics, Eurostat	1992–2021	Deaths: 100<br>Pop.: 100	For visibility reasons, we excluded the remote islands Azores and Madeira.

Slovakia	8 NUTS 3 units (‘kraje’)	Slovakian Statistical Office	1996–2022	Deaths: 100<br>Pop.: 100	–
Slovenia	2 NUTS 2 units (‘kohezijske regije’)	Slovenian Statistical Office	2002–2021	Deaths: 100<br>Pop.: 100	–
Spain	50 NUTS 3 units (‘provincias’)	National Statistics Institute	1990–2021	Deaths: 100<br>Pop.: 85 (after 2001:100)	For visibility reasons, we excluded the Canary Islands.
Sweden	21 NUTS 3 units (‘län’)	Statistics Sweden, Eurostat	1969–2021	Deaths: 100<br>Pop.: 100	–
Switzerland	7 NUTS 2 units (‘Grossregionen’)	Federal Statistical Office	1991–2020	Deaths: 95<br>Pop.: 100	–
United Kingdom	37 NUTS 2 regions	Office for National Statistics (for England & Wales);<br>Human Mortality Database (for Northern Ireland & Scotland)	2002–2021<br>2002–2021<br>1990–2021	England & Wales<br>Deaths: 90<br>Pop.: 90<br>Northern Ireland<br>Deaths: 110<br>Pop.: 110<br>Scotland<br>Deaths: 113<br>Pop: 90	–<br>NUTS 1 level data for Northern Ireland and Scotland due to data quality issues

Supplementary Table 2: Comparison of changes in life expectancy at birth with Schöley et al. (2022) [1].

	Women				Men			
	Schöley et al. 2022		This paper		Schöley et al. 2022		This paper	
**Country**	**2020**	**2021**	**2020**	**2021**	**2020**	**2021**	**2020**	**2021**
Austria	-0,70	-0,70	-0,49	-0,59	-0,99	-1,26	-0,81	-1,12
Belgium	-1,15	-0,33	-0,91	-0,22	-1,29	-0,78	-1,15	-0,70
Switzerland	-0,64	-0,23	-0,90	-0,55	-1,14	-0,74	-1,57	-1,17
Czech Republic	-0,94	-1,76	-0,94	-1,94	-1,25	-2,41	-1,20	-2,60
Germany	-0,32	-0,67	-0,10	-0,52	-0,49	-1,03	-0,30	-0,84
Denmark	-0,03	-0,33	0,00	-0,32	-0,04	-0,17	-0,03	-0,20
Estonia	-0,32	-1,94	-0,07	-1,51	-0,40	-2,65	-0,23	-2,12
Spain	-1,41	-0,93	-1,14	0,02	-1,52	-1,22	-1,38	-0,44
Finland	-0,01	-0,23	-0,02	-0,24	-0,30	-0,32	-0,37	-0,47
France	-0,52	-0,18	-0,43	-0,46	-0,75	-0,53	-0,71	-0,85
North. Ireland	-0,84	-0,95	-0,81	-0,83	-0,79	-0,97	-0,85	-1,05
Scotland	-0,45	-0,69	-0,44	-0,73	-1,07	-1,00	-0,91	-0,83
Hungary	-0,83	-2,20	-0,73	-2,15	-0,93	-2,55	-0,84	-2,68
Iceland	-0,41	-0,39	0,01	0,01	-0,30	-0,11	-0,22	0,04
Italy	-1,07	-0,97	-0,86	-0,76	-1,46	-1,25	-1,33	-1,12
Lithuania	-1,57	-2,97	-1,08	-2,43	-2,25	-2,98	-1,69	-2,58
Netherlands	-0,58	-0,74	-0,52	-0,76	-0,95	-0,94	-0,90	-1,10
Norway	0,04	-0,21	-0,05	-0,19	-0,05	-0,17	-0,26	-0,26
Poland	-1,02	-2,26	-1,02	-2,08	-1,60	-2,64	-1,55	-2,55
Portugal	-0,66	-0,65	-0,59	-0,64	-0,91	-1,02	-0,77	-0,78
Sweden	-0,62	-0,21	-0,30	0,02	-1,01	-0,56	-0,72	-0,31
Slovenia	-1,02	-0,64	-0,87	-0,77	-0,93	-0,88	-1,00	-1,38
Slovakia	-0,94	-2,97	-0,85	-3,09	-1,10	-3,38	-1,07	-3,58

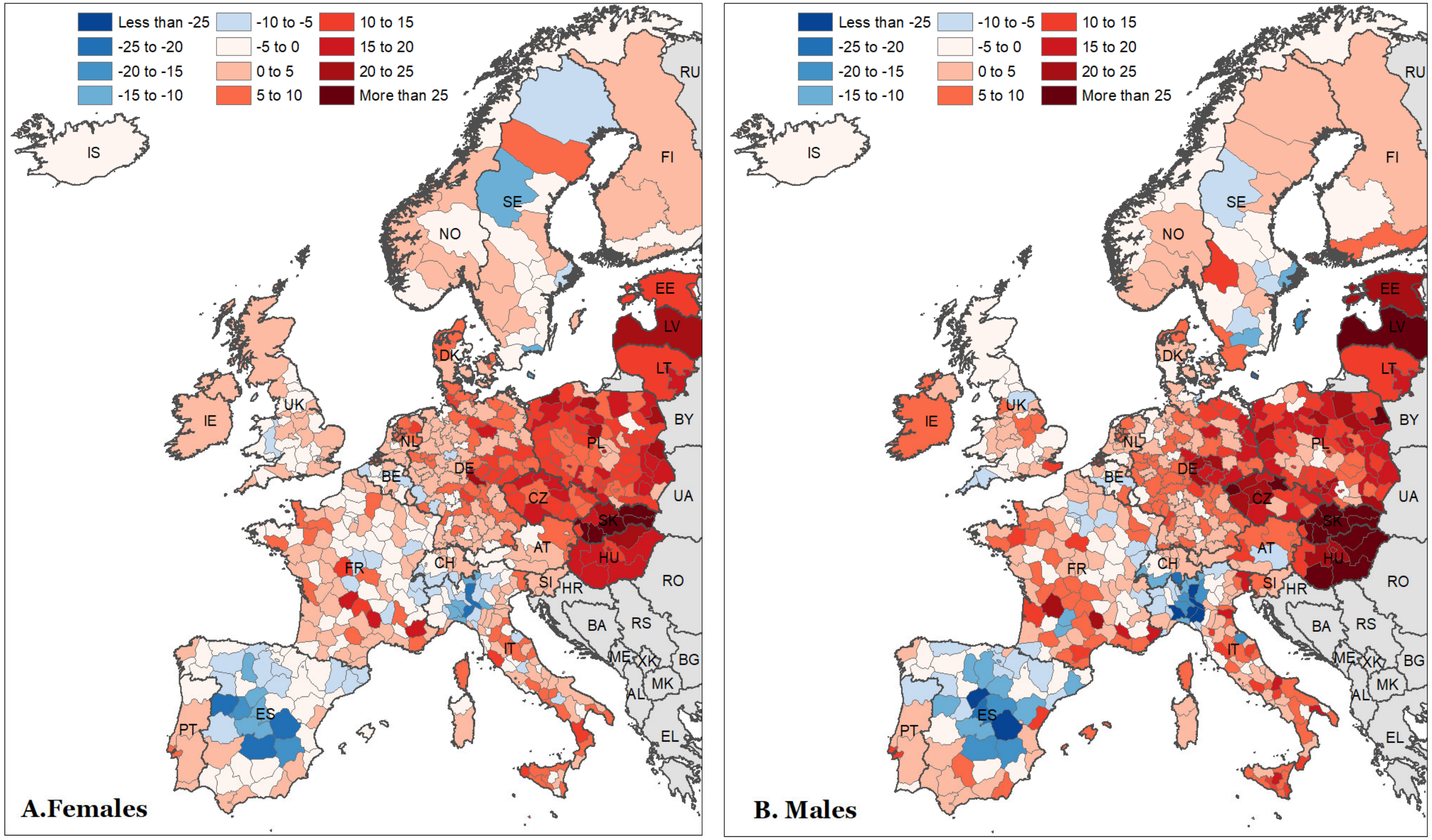

Supplementary Figure 1. Spatial distribution of the change in age-standardized years of life lost (ASYLL) between 2020 and 2021 across 25 European countries

Notes: ASYLL quantifies excess mortality in terms of life years lost. It calculates the potential additional mortality in a given period, associates this age-specific excess mortality with the number of years the population would have lived, and finally sums these values up standardizing them with respect to a reference age structure. Thus, ASYLL is unaffected by the population size and age structure of the underlying population. Age-specific excess mortality is defined as the difference between forecasted mortality rates based on the pre-pandemic mortality trend and the mortality rates observed in the pandemic years 2020 and 2021. Source: Copy from Table A1.

Online supplementary appendix B

A detailed description of the analytic procedure to compute excess mortality is available at:
https://osf.io/fwtsa/?view_only=ba00308358dc4fbaa23de72f9c82d1db

Online supplementary appendix C

Detailed values of our estimates and data visualisation tool are available at:
https://osf.io/fwtsa/?view_only=ba00308358dc4fbaa23de72f9c82d1db

Please read “Online Appendix C.pdf” first.

References

[1] J. Schöley, J. M. Aburto, I. Kashnitsky, M. S. Kniffka, L. Zhang, H. Jaadla, J. B. Dowd et R. Kashyap, «Life expectancy changes since COVID-19,» Nature Human Behaviour, vol. 6, p. 1649–1659, October 2022.
